# A Comprehensive Analysis of Clinical Crowns in Young of Han Nationality with Normal Occlusion Using Intraoral Scanning

**DOI:** 10.1155/2023/2485368

**Published:** 2023-06-05

**Authors:** Rongkai Cao, Piaopiao Qiu, Jianzhao Ni, Hui Xu, Haoxin Pan, Yujie Cao

**Affiliations:** ^1^School & Hospital of Stomatology, Tongji University, Shanghai Engineering Research Center of Tooth Restoration and Regeneration, Shanghai 200072, China; ^2^Department of Stomatology, The First Affiliated Hospital of Fujian Medical University, No. 20, Chazhong Rd, Fuzhou 350004, Fujian, China

## Abstract

**Background:**

The measurement and analysis of clinical crowns play a crucial role in stomatology, anthropology, and studies of genetic and environmental variables in oral and maxillofacial development.

**Purpose:**

The objective of the present study was to measure the parameters of clinical crowns of permanent dentition in youth of Han nationality using intraoral scanning and identify potential influencing factors.

**Materials and Methods:**

A total of 100 subjects (50 males and 50 females) of Han nationality aged 18–24 with normal occlusion were selected. An intraoral scanner was used to obtain the digital dental impressions, and Materialise Magics 21 software was used to measure the mesiodistal diameter (MDD), buccolingual diameter (BLD), height, mesiodistal angle (MDA), and vestibulo-oral angle (VOA) of clinical crowns. The central height was calculated based on the height of clinical crowns. SPSS 27.0 software was used for statistical analysis. The two-independent-sample*t*-test was used to assess discrepancies in clinical crowns between males and females. The paired *t*-test was used to determine differences between antimetric pairs of clinical crowns within the same arch. The repeatability of intraoral scanning was tested using the paired *t*-test between two measurements at one-month intervals. The overall estimated effect was considered significant where *P*  <  0.05.

**Results:**

The MDD, BLD, height, MDA, and VOA of clinical crowns in the youth of Han nationality were measured, and the central height was calculated. No significant difference was found in terms of MDA and VOA between genders and antimetric pairs within the same arch. Regarding the distance parameters, the MDD, BLD, and height of clinical crowns in males were significantly larger than those in females (MDD: U1, U3, U7, L2, L3, L6, and L7: *P* < 0.01; BLD: U1: *P*=0.02; U3–U7 and L1–L7: *P* < 0.01; height: U2: *P*=0.03; and U1, U3–U7, and L3–L7: *P* < 0.01). No significant difference was found in clinical crowns between antimetric pairs within the same arch. Intraoral scanning demonstrated good repeatability in the measurement of clinical crowns.

**Conclusions:**

Apart from MDA and VOA, the parameters of clinical crowns in males were significantly larger than in females. Antimetric pairs of clinical crowns within the same arch demonstrated similar tooth dimensions. In future clinical practice and scientific research in the oral and maxillofacial region, a comprehensive design of sexual and ethnic characteristics should be considered.

## 1. Introduction

The measurement and analysis of clinical crowns hold a significant value in stomatology, anthropology, and studies of genetic and environmental variables in oral and maxillofacial development. The length of the clinical crown, for instance, is of great significance in the functional aesthetic display [1]. Accurate diagnosis of the morphology of clinical crowns is essential for dental healthcare providers to decide on the planning of restoration of tissue defects [2]. Moreover, the precise positioning of brackets at the midpoint of the clinical crown is considered the most critical influential factor for the success of the widely used straight-wire appliances in orthodontics [3]. However, most straight-wire appliances used in clinical practice rely on the normal occlusion data derived from the Caucasian population [4], despite reported discrepancies in clinical crowns among different races, regions, and genders. A previous systematic review and meta-analysis summarized the tooth crown mesiodistal measurements of 6481 participants and demonstrated a small degree of sexual dimorphism existing in all human teeth [5]. Another review evaluated the pancontinental variations in the crown dimensions and found that the disparity in crown dimensions was evident between populations and genders [6]. Given these differences in ethnicity, the existing modalities of straight-wire appliances may not be suitable for Chinese patients. Hence, measuring the clinical crowns of Chinese Han nationality is of great significance to provide a reference for clinical practice in stomatology.

Making dental impressions, typically considered the first step during clinical procedures, is critical for further analysis of clinical crowns. However, traditional dental impressions captured using alginate or silicone rubber impression materials, which are used to record the dentition and occlusal relationship in most clinical scenarios, can be time-consuming and costly. This is due to the entire process, including impression-taking, model fabrication, and model storage [7]. Additionally, an unsatisfactory precision of conventional dental impressions has been shown in the literature because of potential flaws including the potential distortion and expansion of gypsum casts [8]. Manual measurement using dental vernier calipers can be challenging to obtain three-dimensional (3D) data such as the surface area and volume of clinical crowns.

The continuous expansion of digital technology in dentistry has led to the emergence of digital dental impressions, which may offer a possible solution to the problems associated with conventional impressions and facilitate the accurate analysis of clinical crowns. Digital dental impressions have shown promising advantages compared to traditional ones, including real-time imaging, reduced requirements for impression materials, cost-effectiveness, and better communication [9]. With the use of an intraoral scanner, dentists can scan the dentition and obtain occlusion relationships in a 3D manner. Intraoral scanning simplifies the workflow, reduces the operation time, and increases patient comfort compared to conventional methods [10]. In terms of accuracy, data acquired by intraoral scanning are comparable to conventional methods for the fabrication of prostheses [11]. A previous review summarized the difference in orthodontic measurements between digital dental impressions and plaster ones and found that the absolute mean differences were minor and clinically insignificant, indicating the use of digital dental impressions as an alternative to conventional measurement [12]. Studies have demonstrated that the accuracy of intraoral scanning can be acceptable during the clinical practice of dentistry compared to conventional methods [13, 14].

This study aimed to measure and analyzes the parameters of clinical crowns of permanent dentition in youth of Han nationality on the basis of the digital dental impressions obtained by intraoral scanning and identify any potential influencing factors. In addition, this study aims to provide a more reliable reference for tooth dimensions in youth of Han nationality and assist in periodontal, restorative, and orthodontic treatment in clinical settings. The study's null hypothesis was that there existed statistical differences in the parameters of clinical crowns in terms of antimetric pairs within the same arch and different genders.

## 2. Materials and Methods

This study was approved by the Institutional Review Board of the First Affiliated Hospital of Fujian Medical University, Fujian, PR China (No. MRCTA, ECFAH of FMU [2022] 180). Written informed consent was obtained from 100 college students of Han nationality aged 18–24 with normal occlusion in accordance with previous research studies, including 50 males and 50 females.

Participants that fulfilled the following inclusion criteria were selected: (i) a full permanent dentition of at least 28 teeth aged 18–24; (ii) no dental defects, no restorations, and healthy teeth and periodontal tissue; (iii) normal occlusal relations with opposing molars and premolars in contact in right and left excursions; and (iv) the intercuspal position was stable. The following exclusion criteria were applied: (i) anterior open occlusions absent of anterior guidance contacts and (ii) subjects who had undergone prior occlusal adjustment treatment or treatment for temporomandibular disorder.

**Figure 1:**
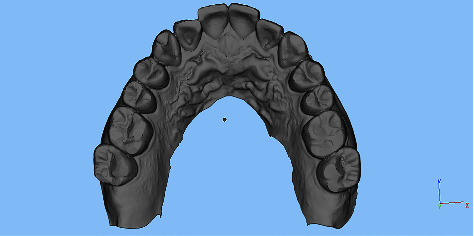
Digital dental impressions obtained by intraoral scanning.

Digital scans of the upper and lower teeth were obtained using an intraoral scanner (Trios color, 3Shape, Denmark). The intraoral scanner's camera was positioned inside the mouth, and the scanning process began at the center of the occlusion with the camera angled at 45 degrees. The scanner then captured continuous data by moving sequentially to the mesial, lingual, distal, and buccal surfaces of the teeth. Images of maxillary and mandibular teeth were acquired separately. The participant was then instructed to bite down with maximum force, and the scanner captured the buccal surfaces to generate the occlusal contacts in the intercuspal position automatically. The resulting scan data were saved in the STL format (Figure 1).

**Figure 2:**
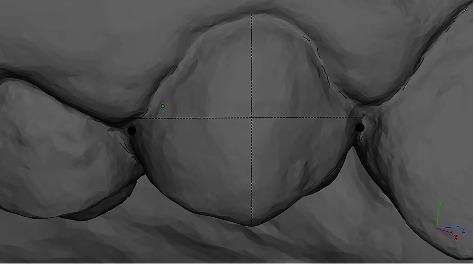
Measurement of mesiodistal diameter of clinical crowns.

**Figure 3:**
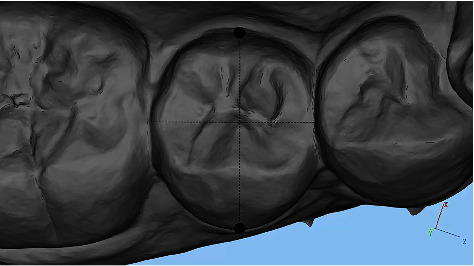
Measurement of buccolingual diameter of clinical crowns.

The scan files obtained from intraoral scanning were imported into the 3D measurement software Materialise Magics 21 (Materialise, Belgium) for data measurement. All tests were conducted in random order by the same trained and experienced examiner. The measurement items included (1) Mesiodistal diameter, which is the maximum vertical distance between the mesial and distal surfaces, also known as the distance between the mesial and distal contact points (Figure 2) and (2) buccolingual diameter, which is the vertical distance between the most convex point of the buccal surface and the lingual surface (Figure 3).

**Figure 4:**
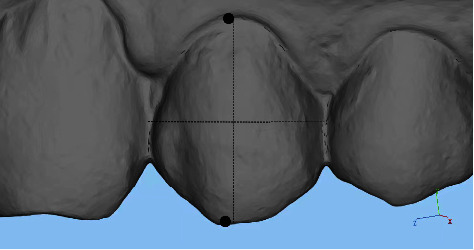
Measurement of height of clinical crowns.

**Figure 5:**
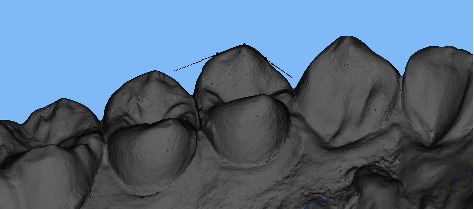
Measurement of the mesiodistal angle of clinical crowns.

**Figure 6:**
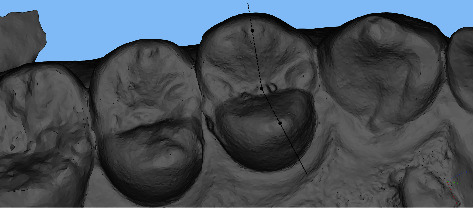
Measurement of the vestibulo-oral angle of clinical crowns.

(3) Height, which is the clinical crown height, was measured along the long axis of the crown. For the incisor, the height is from the midpoint of the incisal edge to the gingival margin. Regarding the canine and bicuspid, clinical crown height refers to the buccal cusp to the gingival margin. As for molars, the molar is from the midpoint of the line between the mesial and distal buccal cusps to the gingival margin (Figure 4). (4) Center height is defined as half of the clinical crown height. (5) The mesiodistal angle is the angle formed by the mesial and distal cusp crests (buccal cusp for premolars and mesiobuccal cusp for molars) (Figure 5). The vestibulo-oral angle is the angle formed by the triangular crest of the buccal tip and the triangular crest of the tongue tip (mesiobuccal cusp for molars) (Figure 6).

Statistical analysis was performed using SPSS software (version 27.0, IBM Corp., Armonk, NY, USA). Descriptive statistics, including mean and standard deviation, were calculated for the measurement data. The discrepancies between clinical crowns of males and females were analyzed using an independent two-sample *t*-test, while the paired *t*-test was used to compare the differences between antimetric pairs of clinical crowns within the same arch. To assess the repeatability of intraoral scanning, 20 subjects were randomly selected for a second measurement after one month, and the paired *t*-test was employed to compare the two measurements. A significance level of *P*  <  0.05 was used to determine the overall estimated effect.

## 3. Results

The MDD, BLD, height, MDA, and VOA of clinical crowns in the youth of Han nationality were measured, and the central height was calculated. The recorded data are listed in Table 1.

The results revealed that in the case of the upper teeth, the sequence of MDD of clinical crowns was U6 > U7 > U1 > U3 > U4 > U2 > U5. While for the lower teeth, the MDD ranked was L6 > L7 > L4 > L5 > L3 > L2 > L1. No significant difference was observed between antimetric pairs of clinical crowns within the same arch for MDD. Nonetheless, notable differences were noted between male and female participants (*P* < 0.05) (Table 2). The sequence of BDD of clinical crowns in the maxillary teeth is the same as that of MDD, which is U6 > U7 > U1 > U3 > U4 > U2 > U5. Regarding the mandible teeth, the first molar presented the largest MDD, whereas the remaining teeth exhibited a decline from posterior to anterior teeth. The BDD of clinical crowns for males was larger than that of females (*P* < 0.05). There was no significant difference between the left and right corresponding teeth (Table 3).

Regarding the maxillary teeth, the clinical crown height was highest for central incisors, followed by canines and lateral incisors, with the height of the remaining teeth decreasing from anterior to posterior. For the lower arch, the clinical crown height measurements were ranked in descending order as follows: L3, L6, L2, L1, L4, L5, and L7. No significant difference was observed in clinical crown height between antimetric pairs of clinical crowns within the same arch. However, the clinical crown height of mandibular molars was higher than that of maxillary molars, and males exhibited a higher clinical crown height than females (*P* < 0.05) (Table 4).

For the upper teeth, the MDA of the mesiobuccal cusp exhibited the largest angle of all measurements, followed by the buccal cusp of the first premolar and the canine cusp. Conversely, the sequence of MDA in the lower teeth was opposite to that of the maxillary teeth, with the canine presenting the largest MDA. For the VOA of the posterior teeth, the VOA of the first molar was greater than that of the first premolar in the upper jaw, whereas the VOA of the first molar was smaller than that of the first premolar in the lower jaw. No significant difference is found in Table 5 and Table 6 terms of MDA and VOA between genders and antimetric pairs within the same arch.

The results of paired *t*-test between two measurements at one-month intervals showed no significant difference in MDD, BLD, MDA, VOA, and height of the clinical crowns, which demonstrated good repeatability of intraoral scanning (*P* > 0.05) (Tables 7 and 8).

## 4. Discussion

Dental crowns can be classified into two types: anatomical crowns and clinical crowns. The anatomical crown, which has been extensively studied due to its ease of measurement, refers to the part of a tooth covered by enamel, with the crown and root demarcated by the tooth neck [15]. On the other hand, the clinical crown refers to the part of the crown exposed in the mouth, which is influenced by the position of the gingival margin. However, measuring and analyzing clinical crowns is complex due to the intricacy of the oral cavity, the challenging process of creating gypsum models, and the difficulty in accurate measurement. Despite these challenges, studying clinical crowns is crucial for both stomatology clinical practice and scientific research related to oral and maxillofacial development. Thus, this study aimed to measure the parameters of clinical crowns of permanent dentition in youth of Han nationality using intraoral scanning and identify potential influencing factors, to provide references for clinical practice and further research of related products for young people. The suggested hypothesis regarding statistical differences of clinical crowns was valid only for distance parameters between males and females, according to the findings of this study.

MDD and BDD are two essential parameters used to measure tooth dimensions. For example, MDD is the basic parameter for further investigation of the tooth index. Prior research has explored the differences in MDD and BDD between genders and antimetric pairs. For instance, Potter et al. have suggested that the ideal left and right teeth should be symmetrical since strong evidence exists for the existence of significant genetic determinants of almost all of the individual tooth dimensions [16]. The results of our study, which measured clinical crowns in young Han individuals, showed no significant difference between antimetric pairs, which is consistent with the literature. Consequently, the mean values of the left and right homonymous teeth were computed in our study. Additionally, Bishara et al. reported differences in MDD and BDD between males and females [17]. Our results corroborate this finding, indicating that the clinical crowns of males are significantly larger than those of females.

Clinical crown height refers to the distance from the most apical concavity of the gingival margin to the occlusal surface or incisal edge of a particular tooth on the facial surface along the long axis of the tooth [18]. Clinical crown height is a critical parameter that can evaluate and determine the position of the gingival margin. Studies have proposed that investigation of clinical crown height should report results by age and tooth type due to their changes with time [19]. Therefore, it is essential to investigate the clinical crown height and center height in the young of Han nationality for the diagnosis and treatment of oral disease and related product development. After comparing the results with that of normal occlusion reported in the literature, differences between nationalities and ages in terms of clinical crown center height were found (Table 9) [20–22]. In addition, results showed significant differences between males and females, while no significant difference was found between the left and right teeth, which are consistent with the results of MDD and BLD.

The distance parameters measured in this study could provide a more reliable reference for research and product development in youth of Han nationality and help periodontal, restorative, and orthodontic treatment in clinical settings. For example, the norms for clinical crown heights are useful in the diagnosis of oral diseases such as gingival recession [23]. In addition, based on the distances mentioned above, other parameters like width/length ratios can be calculated and used for treatment in clinical settings. The final position of the gingival margin during surgery can be determined based on the width/length ratios in conjunction with other clinical parameters. In addition, prosthodontists can determine suitable tooth height in patients that have undergone excessive occlusal wear by using calculations if tooth MDD can be measured [24]. Moreover, in recent years, because of the increased participation of young people in orthodontic treatment and the application of straight-wire appliances, the determination of the reference of the clinical crown center height for the young generation may help adhere to the bracket correctly and improve the treatment effect. Accordingly, the results of this study are of great significance to provide a reference for clinical practice in dentistry.

In dentistry, the relationship between tooth morphology, occlusal relations, and mandibular glenoid fossa morphology is considered a critical issue. The results of this study may provide references for the diagnosis of tooth cracked syndrome and the prevention of temporomandibular joint disorder. The MDA and VOA are two key parameters of dental morphology that demonstrated an underlying relationship with the temporomandibular joint movement [25]. Changes in MDA and VOA can lead to temporomandibular joint disorder, which can cause adaptive reconstruction of the temporomandibular joint. An increase in MDA and a decrease in VOA in right excursions may cause occlusal interference and temporomandibular joint disorder [26]. Furthermore, researchers have proposed that the sagittal condylar inclination (SCI) values should be considered during the prosthodontic treatment of patients with a skeletal discrepancy [27]. VOA and MDA significantly decreased with the increase of SCI under the study models in vitro [25]. In addition, VOA was one of the most critical anatomical parameters in the etiology of cracked tooth syndrome in molars. A previous study found that the mean VOA of the cracked teeth in upper molars was 97.21 ± 9.35, which is much smaller than the VOA of the control group (114.55 ± 7.37) [28]. Another study also concluded that the VOA in the cracked tooth group was smaller than that in the normal group [29]. The results of this study were in accordance with the normal group of the previous study [28, 29]. Moreover, our study found no significant difference in MDA and VOA between genders and antimetric pairs within the same arch, which is consistent with the conclusion proposed by Potter et al. [16].

Conventionally, dental impressions were obtained with silicone rubber or alginate impression materials, and dental morphology was performed through contact measurement using calipers and protractors [30]. However, due to the complexity of 3D anatomy in clinical crowns, accurately and efficiently measuring dental morphology has been challenging. In this study, an intraoral scanner was used to obtain digital dental impressions of the subjects, and the parameters of clinical crowns were measured using software. This digital process eliminates potential errors in model manufacturing and contact measurement and reduces clinical time. Previous research has reported that there is no significant difference between intraoral scanning and plaster models, and digital dental impressions can meet clinical requirements [31–33]. The results of the paired *t*-test at one-month intervals showed no significant difference in all measured parameters, which also demonstrated the repeatability of intraoral scanning. Additionally, the selected subjects improve the reliability of this study, as their recently erupted permanent teeth and relatively less abrasion reduce the potential errors of clinical crown measurement and analysis. Moreover, with the continuous expansion of digital technology, multiple innovative devices have been applied in dentistry, including different kinds of lasers, ozonization machines, and electronic-controlled anesthesia. As a growing industry in dental medicine, ozone therapy shows much potential in the treatment of many disorders such as sensitivity and root canal therapy [34]. The use of computer-controlled local anesthetic delivery systems also presents the advantages of controlling the speed of the anesthetic injected into tissues, which could be an alternative to the traditional syringe to reduce pain during the anesthetic procedure [35]. Photodynamic therapy in association with nonsurgical periodontal treatment could improve the clinical parameters at 3 months [36]. The innovative devices mentioned above significantly simplify the clinical processes, raise the quality of treatment, and improve patient satisfaction.

The present study has several limitations that should be considered. First, this study only measured and analyzed the clinical crowns in young individuals of Han nationality, while China has a diverse population with different ethnicities. Therefore, the results may not apply to other ethnic groups. Second, the number of subjects obtained in this study was in accordance with the previous research studies similar to this article. Accordingly, the sample size of this study may be relatively small, and a larger sample size may be obtained to improve the reliability of the results. Future studies should focus on a larger sample size to explore the differences in clinical crowns among various nationalities and regions, to better determine the parameters of clinical crowns in specific populations and help improve clinical practice and related research.

## 5. Conclusions

Using an intraoral scanner, the present study measures and analyzes the morphology of the clinical crown in youth of Han nationality and identifies the potential influencing factors. Apart from MDA and VOA, an obvious difference was found in clinical crowns between males and females. The MDD, BLD, and height of clinical crowns in males were significantly larger than in females. No significant difference was found in clinical crowns between antimetric pairs within the same arch. Results of the present study provide a more reliable reference for tooth anatomy in youth of Han nationality and help in periodontal, restorative, and orthodontic treatment in clinical settings. In the future, during the clinical practice of stomatology, a comprehensive design of sexual and ethnic characteristics should be considered.

## Figures and Tables

**Table 1:** Measurement of clinical crowns of permanent teeth in the youth of Han nationality.

	Mesiodistal diameter	Buccolingual diameter	Height	Center height
U1	8.48 ± 0.52	7.13 ± 0.61	9.58 ± 0.96	4.79 ± 0.48
U2	7.06 ± 0.63	6.46 ± 0.67	8.19 ± 0.95	4.10 ± 0.48
U3	8.00 ± 0.57	8.15 ± 0.77	9.11 ± 1.11	4.56 ± 0.56
U4	7.45 ± 0.51	9.51 ± 0.63	7.76 ± 0.81	3.88 ± 0.41
U5	6.95 ± 0.45	9.42 ± 0.60	6.65 ± 0.86	3.33 ± 0.43
U6	10.56 ± 0.61	11.19 ± 0.67	6.19 ± 0.90	3.10 ± 0.45
U7	9.99 ± 0.68	11.26 ± 0.74	5.88 ± 0.79	2.94 ± 0.39
L1	5.36 ± 0.40	5.91 ± 0.53	7.71 ± 0.93	3.86 ± 0.47
L2	5.97 ± 0.46	6.37 ± 0.48	8.10 ± 0.81	4.05 ± 0.40
L3	6.88 ± 0.51	7.76 ± 0.62	9.49 ± 1.14	4.75 ± 0.57
L4	7.25 ± 0.53	8.07 ± 0.58	8.39 ± 0.79	4.20 ± 0.39
L5	7.24 ± 0.51	8.41 ± 0.55	7.25 ± 0.74	3.63 ± 0.37
L6	11.12 ± 0.88	10.68 ± 0.62	6.58 ± 0.50	3.29 ± 0.25
L7	10.39 ± 0.74	10.39 ± 0.64	6.08 ± 0.78	3.04 ± 0.39

**Table 2:** Analysis of difference of mesiodistal diameter of clinical crowns of permanent teeth in the youth of Han nationality.

	Left	Right	*P*	Male	Female	*P*
U1	8.47 ± 0.52	8.49 ± 0.52	0.45	8.59 ± 0.55	8.38 ± 0.47	<0.01
U2	7.05 ± 0.62	7.06 ± 0.64	0.84	7.10 ± 0.65	7.02 ± 0.61	0.36
U3	8.01 ± 0.57	7.98 ± 0.57	0.40	8.10 ± 0.53	7.90 ± 0.58	0.01
U4	7.44 ± 0.54	7.47 ± 0.49	0.25	7.50 ± 0.52	7.41 ± 0.51	0.24
U5	6.97 ± 0.46	6.93 ± 0.44	0.16	6.95 ± 0.45	6.95 ± 0.44	0.99
U6	10.59 ± 0.63	10.53 ± 0.60	0.13	10.64 ± 0.60	10.48 ± 0.62	0.07
U7	10.01 ± 0.67	9.98 ± 0.68	0.54	10.14 ± 0.64	9.85 ± 0.69	<0.01
L1	5.35 ± 0.39	5.37 ± 0.41	0.45	5.39 ± 0.38	5.33 ± 0.43	0.29
L2	6.00 ± 0.45	5.95 ± 0.47	0.08	6.07 ± 0.40	5.88 ± 0.50	<0.01
L3	6.86 ± 0.47	6.89 ± 0.55	0.16	6.98 ± 0.52	6.77 ± 0.48	<0.01
L4	7.25 ± 0.53	7.25 ± 0.53	0.97	7.31 ± 0.56	7.19 ± 0.49	0.10
L5	7.24 ± 0.52	7.23 ± 0.51	0.80	7.31 ± 0.57	7.17 ± 0.44	0.06
L6	11.11 ± 1.10	11.12 ± 0.61	0.91	11.32 ± 0.63	10.92 ± 1.04	<0.01
L7	10.38 ± 0.77	10.40 ± 0.71	0.79	10.54 ± 0.78	10.23 ± 0.66	<0.01

**Table 3:** Analysis of the difference of buccolingual diameter of clinical crowns of permanent teeth in the youth of Han nationality.

	Left	Right	*P*	Male	Female	*P*
U1	7.10 ± 0.57	7.16 ± 0.64	0.20	7.23 ± 0.59	7.03 ± 0.60	0.02
U2	6.48 ± 0.68	6.44 ± 0.66	0.28	6.48 ± 0.70	6.43 ± 0.64	0.60
U3	8.15 ± 0.80	8.16 ± 0.76	0.91	8.34 ± 0.83	7.96 ± 0.67	<0.01
U4	9.50 ± 0.63	9.53 ± 0.63	0.32	9.65 ± 0.66	9.38 ± 0.57	<0.01
U5	9.43 ± 0.61	9.42 ± 0.60	0.28	9.54 ± 0.66	9.30 ± 0.51	<0.01
U6	11.19 ± 0.70	11.18 ± 0.64	0.68	11.40 ± 0.69	10.97 ± 0.57	<0.01
U7	11.25 ± 0.76	11.27 ± 0.73	0.67	11.48 ± 0.74	11.04 ± 0.69	<0.01
L1	5.92 ± 0.55	5.90 ± 0.52	0.52	6.03 ± 0.60	5.78 ± 0.42	<0.01
L2	6.37 ± 0.48	6.37 ± 0.50	0.93	6.50 ± 0.48	6.24 ± 0.46	<0.01
L3	7.77 ± 0.61	7.75 ± 0.64	0.47	7.96 ± 0.67	7.57 ± 0.51	<0.01
L4	8.06 ± 0.56	8.08 ± 0.60	0.43	8.20 ± 0.60	7.93 ± 0.53	<0.01
L5	8.40 ± 0.55	8.43 ± 0.54	0.44	8.51 ± 0.57	8.32 ± 0.51	0.01
L6	10.68 ± 0.63	10.68 ± 0.61	0.86	10.85 ± 0.65	10.51 ± 0.53	<0.01
L7	10.37 ± 0.61	10.41 ± 0.66	0.18	10.60 ± 0.63	10.17 ± 0.56	<0.01

**Table 4:** Analysis of the difference of height of clinical crowns of permanent teeth in the youth of Han nationality.

	Left	Right	*P*	Male	Female	*P*
U1	9.57 ± 0.96	9.60 ± 0.96	0.46	9.85 ± 0.98	9.32 ± 0.86	<0.01
U2	8.14 ± 0.97	8.25 ± 0.94	0.08	8.34 ± 1.00	8.05 ± 0.89	0.03
U3	9.09 ± 1.14	9.13 ± 1.09	0.31	9.35 ± 1.19	8.88 ± 0.98	<0.01
U4	7.72 ± 0.81	7.80 ± 0.82	0.13	7.94 ± 0.86	7.58 ± 0.72	<0.01
U5	6.64 ± 0.85	6.66 ± 0.87	0.65	6.84 ± 0.86	6.46 ± 0.82	<0.01
U6	6.18 ± 0.91	6.20 ± 0.90	0.62	6.42 ± 0.93	5.96 ± 0.81	<0.01
U7	5.83 ± 0.80	5.93 ± 0.78	0.07	6.12 ± 0.77	5.64 ± 0.73	<0.01
L1	7.70 ± 0.92	7.72 ± 0.95	0.64	7.82 ± 1.10	7.61 ± 0.72	0.12
L2	8.09 ± 0.79	8.11 ± 0.83	0.73	8.18 ± 0.91	8.02 ± 0.69	0.17
L3	9.45 ± 1.13	9.53 ± 1.15	0.11	9.78 ± 1.25	9.21 ± 0.93	<0.01
L4	8.35 ± 0.78	8.42 ± 0.80	0.18	8.66 ± 0.77	8.11 ± 0.71	<0.01
L5	7.23 ± 0.67	7.25 ± 0.79	0.67	7.43 ± 0.67	7.07 ± 0.76	<0.01
L6	6.58 ± 0.52	6.58 ± 0.49	0.91	6.71 ± 0.52	6.46 ± 0.46	<0.01
L7	6.09 ± 0.81	6.08 ± 0.76	0.80	6.23 ± 0.77	5.94 ± 0.77	0.01

**Table 5:** Analysis of the difference of the mesiodistal angle of clinical crowns of permanent teeth in the youth of Han nationality.

	Left	Right	*P*	Male	Female	*P*
U3	127.93 ± 10.19	127.33 ± 10.10	0.374	127.78 ± 10.10	127.48 ± 10.20	0.834
U4	131.89 ± 7.66	130.79 ± 7.50	0.088	130.68 ± 7.65	132.00 ± 7.50	0.218
U6	137.33 ± 8.25	136.40 ± 8.46	0.053	137.20 ± 7.92	136.52 ± 8.78	0.566
L3	129.94 ± 9.36	129.58 ± 9.33	0.345	129.91 ± 9.19	129.61 ± 9.50	0.303
L4	130.72 ± 6.94	130.14 ± 6.92	0.075	130.51 ± 6.09	130.35 ± 7.69	0.864
L6	139.28 ± 11.82	138.25 ± 12.75	0.117	137.39 ± 12.60	140.14 ± 11.85	0.113

**Table 6:** Analysis of the difference of the vestibulo-oral angle of clinical crowns of permanent teeth in youth of Han nationality.

	Left	Right	*P*	Male	Female	*P*
U4	108.67 ± 10.28	107.72 ± 10.55	0.100	108.88 ± 11.42	107.52 ± 9.28	0.356
U6	114.01 ± 10.66	113.70 ± 10.92	0.683	114.25 ± 11.75	113.46 ± 9.73	0.605
L4	133.89 ± 12.72	132.65 ± 12.77	0.086	132.90 ± 13.01	133.64 ± 12.50	0.681
L6	116.43 ± 13.64	117.56 ± 14.47	0.172	116.43 ± 14.86	117.56 ± 13.21	0.571

**Table 7:** The repeatability analysis of distance parameters of clinical crowns of permanent teeth in the youth of Han nationality.

	Mesiodistal diameter			Buccolingual diameter			Height		
	First	Second	*P*	First	Second	*P*	First	Second	*P*
U1	8.72 ± 0.46	8.70 ± 0.47	0.29	7.36 ± 0.56	7.36 ± 0.58	0.97	9.87 ± 0.78	9.83 ± 0.78	0.15
U2	7.35 ± 0.59	7.35 ± 0.64	0.81	6.86 ± 0.62	6.84 ± 0.65	0.36	8.36 ± 0.85	8.36 ± 0.84	0.69
U3	8.33 ± 0.47	8.34 ± 0.51	0.66	8.15 ± 0.72	8.17 ± 0.73	0.07	9.09 ± 0.98	8.98 ± 1.09	0.94
U4	7.74 ± 0.51	7.70 ± 0.55	0.07	9.62 ± 0.64	9.20 ± 2.00	0.37	7.86 ± 0.74	7.85 ± 0.74	0.88
U5	7.20 ± 0.50	7.21 ± 0.49	0.54	9.53 ± 0.61	9.53 ± 0.61	0.93	6.73 ± 0.61	6.71 ± 0.66	0.27
U6	10.92 ± 0.62	10.93 ± 0.59	0.69	11.14 ± 0.66	11.13 ± 0.64	0.86	6.41 ± 0.72	6.48 ± 0.76	0.33
U7	10.21 ± 0.92	10.21 ± 0.87	0.94	11.33 ± 0.76	11.38 ± 0.81	0.08	6.24 ± 0.55	6.31 ± 0.52	0.07
L1	5.51 ± 0.40	5.50 ± 0.40	0.5	6.12 ± 0.43	6.14 ± 0.38	0.23	7.74 ± 0.76	7.70 ± 0.77	0.06
L2	6.13 ± 0.34	6.15 ± 0.35	0.46	6.64 ± 0.39	6.61 ± 0.38	0.18	8.16 ± 0.67	8.15 ± 0.72	0.53
L3	7.17 ± 0.40	7.15 ± 0.37	0.24	7.99 ± 0.61	7.97 ± 0.64	0.55	9.95 ± 1.06	9.96 ± 1.05	0.88
L4	7.46 ± 0.50	7.43 ± 0.49	0.23	8.19 ± 0.64	8.23 ± 0.63	0.15	8.67 ± 0.78	8.64 ± 0.75	0.1
L5	7.47 ± 0.46	7.44 ± 0.46	0.07	8.56 ± 0.61	8.64 ± 0.69	0.1	7.47 ± 0.62	7.46 ± 0.58	0.69
L6	11.22 ± 0.54	11.22 ± 0.58	0.9	10.64 ± 0.62	10.69 ± 0.63	0.06	6.91 ± 0.51	6.94 ± 0.53	0.28
L7	10.61 ± 0.83	10.59 ± 0.81	0.48	10.41 ± 0.71	10.45 ± 0.71	0.1	6.11 ± 0.72	6.32 ± 1.08	0.26

**Table 8:** The repeatability analysis of angle parameters of clinical crowns of permanent teeth in the youth of Han nationality.

	Mesiodistal angle			Vestibulo-oral angle		
	First	Second	*P*	First	Second	*P*
U3	130.09 ± 6.96	129.42 ± 6.08	0.27	N/A	N/A	N/A
U4	132.22 ± 5.78	132.15 ± 5.50	0.90	110.31 ± 10.27	110.02 ± 9.80	0.65
U6	139.25 ± 6.96	138.35 ± 7.25	0.06	117.43 ± 9.12	116.96 ± 9.06	0.48
L3	128.72 ± 8.63	128.52 ± 8.79	0.73	N/A	N/A	N/A
L4	129.95 ± 7.60	129.52 ± 7.42	0.35	134.26 ± 12.18	134.41 ± 11.89	0.79
L6	144.42 ± 8.66	144.26 ± 9.07	0.72	117.98 ± 18.64	117.93 ± 18.92	0.92

**Table 9:** Comparison of measurement of the center height of clinical crowns.

Studies	Population	U1	U2	U3	U4	U5	U6	U7	L1	L2	L3	L4	L5	L6	L7
This study	Han	4.8	4.1	4.6	3.9	3.3	3.1	2.9	3.9	4.1	4.8	4.2	3.6	3.3	3.0
Liu et al. [20]	Mongolia	4.9	4.2	4.8	3.9	3.3	3.1	2.9	4.2	4.2	4.6	4.1	3.5	3.2	3.0
Mi et al. [21]	Salar	4.5	3.8	4.3	3.7	3.0	2.9	2.8	3.9	4.0	4.6	3.9	3.3	3.2	2.9
Palone et al. [22]	Italian	5.1	4.2	4.8	4.1	3.6	3.0	2.7	4.0	4.2	4.9	4.3	3.9	3.4	2.9
Palone et al. [22]	Mozambican	4.8	4.2	4.8	4.0	3.4	2.6	2.3	3.9	4.1	5.0	4.2	3.7	2.9	2.5

## Data Availability

The data used to support this study are available from the corresponding author upon request.
